# The impact of income gap on the inverted U-shaped total factor productivity and its mechanisms: Evidence from transnational-level analysis

**DOI:** 10.1371/journal.pone.0228023

**Published:** 2020-01-28

**Authors:** Weiwei He, Yabin Zhang, Yuan Zhong, Juanjuan Chen

**Affiliations:** 1 School of Economics and Trade, Hunan University, Changsha, Hunan, China; 2 Business School, Hunan Normal University, Changsha, Hunan, China; The Bucharest University of Economic Studies, ROMANIA

## Abstract

This paper attempts to verify the “inverted U” effect of the income gap on the total factor productivity (TFP) of the country or region by constructing a theoretical model that includes income effects and substitution effects. Based on this, this paper uses the multinational panel data of 53 countries in the world from 1995 to 2014 for empirical research. The research shows that: (1) the income gap has an “inverted U-shaped” effect on the national TFP, and the inflection point is located at about 0.269; (2) The income gap is also verified by the “inverted U” influence mechanism of the national TFP by affecting the income effect and the substitution effect; (3) The difference in the development stage determines that countries should adopt an income distribution policy that is compatible with their own country. The conclusions of this paper strengthen the confidence and determination to improve the supply system and promote regional innovation, and confirm the necessity of promoting global economic restructuring and achieving global inclusive development to a certain extent.

## 1. Introduction

Since the outbreak of the industrial revolution, scientific and technological progress has promoted the world economy to achieve long-term and high-speed growth. After the Second World War, technological progress has become the main engine of economic growth in the world. Under the conditions of profound changes in the global pattern, the world economy presents two new characteristics: the explosive growth of global production efficiency and the significant change in income gap. As a result, the sustainability and desirability of global economic growth are increasingly questioned by two factors: first, the deepening of the global trade pattern and the division of labor system have led to major changes in the current international supply and demand pattern of resources. The key factor which affects the economic development of various regions of the world is the international resource supply and demand relationship formed by the current international resource supply and demand pattern characterized by the scarcity of resources and their high prices (Li Qiang, 2014)[1]. Therefore, the relative cost of land, labor, energy and other factors varies greatly between regions, so that improving the efficiency of factors will play a more significant role in global inclusive growth. In particular, the phenomenon of labor shortage and the significant trend of increasing in labor costs has been highlighted in some countries and regions. Countries and regions have to face the arrival of the “Lewis Turning Point” (Cai Fang, 2013) [2], and the increasing productivity in various regions, especially in developing countries, can significantly promote the potential for long-term economic growth (Chai Honghui,2017) [3]. Thus, the combination of input and output efficiency measured by technological progress will become a new impetus for global economic growth.

The second factor is that the trade globalization and technology diffusion have led to the differentiation of wages between skilled and unskilled workers (Feenstra and Hanson, 2000; Bustos, 2011) [4–5]. Both are contrary to the current era of “mutual benefit, coordinated development”. The economic situation and the development stages varies from region to region. Therefore, maintaining high economic growth, narrowing the income gap, promoting equal opportunities, and achieving “inclusive growth” in the global economy are the overall welfare for the people from all over the world. Considering the key position of each region and its internal income distribution in the world distribution pattern, in the middle to late industrialization where scale efficiency continues to decline and major factor productivity increases, industry development at maturity and decay depends on the needs of the middle and low income groups. Therefore, narrowing the income distribution gap and increasing the income of the middle and low-income groups is an important way to stabilize economic growth (Zhang Laiming and Li Jianwei, 2016) [6].

Then, what kind of interaction between technological progress and income disparity becomes the key issue. From the logical point of view, technological progress is the decisive factor that currently affects the stability and growth of the world economy, while the income gap reflects the difference in the distribution of economic growth results among various society members. So, there must be a natural connection between the income gap and the technological changes in the process of continuous economic operation. Particularly, it is important to note that technological progress is the most central factor in the transformation of the world’s economic growth model at present, so the problem is obvious: will the income gap have an impact on technological progress? Chen Honglei and Yan Weifang, Fan Jianshuang, etc. all believe that ignoring the income gap will overestimate the regional technology level and emphasize the positive impact of narrowing the urban-rural income gap on technological progress [7–8]. If the answer is yes, then what is the way and mechanism of its influence? In order to answer the above questions, based on the literature review, this paper draws on the logical framework of Gao Fan and Wang Yanan “The Urban-Rural Income Gap Affects the Inverted U-Shaped Hypothesis of TFP” and makes improvements as follows: first, trying to explore a new model if regional economic growth from the perspective of inclusive growth; The second is to expand the research scope into the level of world economy, and use the panel data of 53 countries in the world from 1995 to 2014 for quantitative analysis, from which we can reveal the mechanism of the impact of income gap exerted on technological progress, and try to make a useful complement to existing research and propose the response policies[9].

## 2. Literature review

As a core issue of economic research, the issue of income distribution has extensive links with macroeconomics and microeconomics. Forbes pointed out that the income gap is conducive to economic growth in the short term, but detrimental in the long run [10]. This view is supported by other similar literature (Wang Shaoping and Ouyang Zhigang, 2008; Liao Xinlin et al. 2012) [11–12]. However, if technological progress is seen as a key driver of economic development, the above literature rarely studied the interaction between income distribution and technological progress or production efficiency. In recent years, some studies have filled the above-mentioned "vacuum zone" to a certain extent. Research on the impact of technological advances on the income gap has been carried out earlier. Kats & Autor and Card & Dinardo argued that skill-biased technical change is an important cause of wage differentiation between skilled and unskilled workers [13–14]. On the basis of this, Acemoglu pointed out that the direction of technological advancement dominated by relative prices is an important reason for the improvement of skill-biased technology, and this view has been supported by a series of studies. (Robortson, 2000; Pinelopi Koujianou Goldberg and NinaPavcnik, 2003; Xu Haiping and Wang Yuelong, 2010; Yuan Zhigang and Xie Dongdong, 2011) [15–19]. However, such studies have simultaneously focused on the impact of technological advances on the income gap, but have neglected the impact of the income gap on technological progress.

However, in recent years, some of the literature’s ideas are significantly different from the previous ones. The research theme is determined as the impact of the income gap on technological progress. Its core framework is to explore how the income gap affects technological progress from the perspective of product as well as labor supply and demand respectively. Murphy, Shriver and Vinshny argued that the income gap in the context of heterogeneity in consumer preferences affects technological innovation in enterprises and thus affects the level of regional industrialization [20]. Zweimuller, Zeimuller and Brunner extended Murphy’s model to say that the narrowing of the income gap will increase the market demand for high-quality products due to the hierarchic preferences of consumers [21–22]. Subsequently, Foellmi and Zweimuller clarified the dual impact of the income gap on technological progress based on the product heterogeneity model [23]. Higher income gaps may lead companies to obtain higher prices for innovative products on the one hand and smaller markets on the other. While theoretical studies have shown that the price effect of the income gap often exceeds the market size effect. Therefore, the widening income gap in the context of low income distribution inequality may have a positive effect on innovation and growth. The viewpoint above has also been supported by many research conclusions. Vona and Patriarca pointed out from the perspective of environmental technology innovation that the degree of income gap has a non-linear effect on the relationship between technological innovations through the mediating effect of market demand [24]. An Tongliang, Qian Huixiong, Li Zilian, and Zhu Jiangli emphasized that the impact of income gap on technological innovation is achieved by changing the structure and quantity of demand [25–26]. Li Jingrui, according to the study of cross-country data on income disparities, believed that the income gap will affect product innovation through local market demand [27].

The studies above are important for understanding the issue of income disparity affecting regional TFPs. First, the empirical conclusions on the impact of income distribution on economic growth in a country are quite different. Some scholars believe that the widening income gap will benefit economic growth in the short term(Li and Zou, 1998; Forbes, 2000)[28–29]. While other scholars believe that the income gap has a negative effect on economic growth (Fishman and Simhon, 2002; Wan, Lu and Chen, 2006; Galor, Moav and Vollrath, 2009) [30–32]. Some scholars have pointed out that there is uncertainty about the impact of income disparity on economic growth (Voitchovsky, 2005): maybe it is conducive to growth in the short term, but not conducive to growth in the long run (Barro,1999; Chambers and Krause,2010) [33–35]. Secondly, the existing literature attempts to explore the trends and potential influencing factors of TFP in different regions from different perspectives. These factors mainly refer to trade openness (Miller and Uphadhyay, 2000), human capital (Yan Pengfei and Wang Bing, 2004), and spatial gathering (Zhao Wei, Zhang Cui, 2008) [36–38]. Li Jingrui and Deng Xiaofeng empirically studied how the moderate-income gap stimulates technological innovation through the panel data of the BRICS countries from 2000 to 2011[39]. Liu Run, Song Yu used the panel data of 19 middle-income countries in the world from 1996 to 2006 to find that the expansion of income gap at this stage will inhibit technological innovation [40]. Cheng Wen and Zhang Jianhua further pointed out through model derivation and numerical simulation that the different stages of economic development in different regions have led to a distinctive impact on the independent innovation behavior of domestic enterprises [41].

The discussion on how the income gap of a country affects the domestic TFP needs to be improved from many perspectives. First, existing research tends to stick to a single “demand” perspective to examine the impact of income disparities on technological innovation, which is obviously not consistent with the context of the shrinking world demand caused by the international financial crisis. Gao Fan, Wang Yanan analyzes the impact mechanism of income gap on technological progress in two dimensions: comprehensive supply and demand [42]. They provide a new idea for examining the impact of income gap exerting on the technical level of the region. Second, existing researches often explain the relationship between income gap and technological innovation as a linear relationship. But combined with the non-linear relationship between income disparity and economic growth in the theory of distribution as well as the positive correlation between technological innovation and economic growth in growth theory, there may also be a non-linear correlation between the impact of income disparity and technological innovation.

Finally, the existing research on the subject of the survey often has a tendency of “convergence”. However, considering the significant differences in the development level, factor level and environmental level of each region, the impact of the income gap on technological innovation in the region needs to effectively distinguish the actual differences in the development stage of the region. Meanwhile, spatial correlation should also be considered in the study, that is, the introduction of spatial econometric models to discuss the impact of income disparity on domestic TFP. Therefore, this paper attempts to improve the theoretical model of urban-rural income gap constructed by Gao Fan and Wang Yanan (2016) [9]. On this basis, from the two-dimensional perspective of the substitution effect and income effect of the supply and the consumption, this paper trys to raise the “U”-shaped hypothesis that the income gap affects the domestic TFP; and uses the panel data as well as spatial measurement models of 53 countries in the world from 1995 to 2014 for empirical research; finally, combined with theoretical and empirical analysis, proposes the policy recommendations for improving TFP in various regions.

## 3. Impact mechanism

### 3.1 The inverted "U" type hypothesis between income gap and TFP

According to the above, TFP is the result of a cumulative accumulation of many factors. While under the premise of other factors, TFP is positively related to labor quality and human capital investment, and is positively related to high quality products and market demand. Labor supply and product demand are the result of self-optimized configuration based on market mechanism, both of which reflect the stage characteristics of the income distribution pattern of the economy in the process of economic development. In addition, as a micro-subject that promotes technological advancement and productivity improvement, the market has an impact on TFP through the “intermediary channels” of the supply and consumption. Therefore, this paper will propose two theoretical core mechanisms for the income gap affecting TFP.

### 3.2 Consumption mechanism: Income effect and substitution effect

The income gap will cause TFP changes by affecting total consumption, and this effect can be subdivided into two types of effects: first, the consumption effect. TFP reflects the overall productivity level of many related companies. Innovative enterprises rely on innovative products to obtain excess profits, which in turn causes other companies to follow and lead to the dependence of the expansion of innovation production on the expansion of market scale caused by consumption expansion. Chen Fenglong and Xu Kangning, Feng Wei and Yan Jiatao all did empirical research using China’s provincial panel data and found that the improvement of average domestic income level has promoted technological progress and TFP since the 21st century in the country [43–44]. According to the above, the excessive income gap may have a negative impact on TFP growth by curbing consumption expansion. The reason is that according to the traditional consumption theory, the consumer’s marginal propensity to consume decreases as the income level increases. Therefore, an excessively high-income gap will lower the total social consumption propensity and reduce the total consumption scale. Conversely, Han Liyan and Du Chunyue (2012) found through empirical tests that the decline in income gap has significantly increased the per capita disposables of residents and promoted the growth of household consumption [45]. On this basis, Yang Yiwen and Zheng Jianghuai (2103), Jin Xiaotong and Huang Rui (2017) also pointed out that market demand expansion has positive significance for stimulating enterprise innovation and improving production efficiency [46–47]. Therefore, it can be considered that decline in the income gap will encourage companies to increase production efficiency by stimulating a total consumption upgrade to promote a country’s TFP level.

Empirical studies in 19 countries show that the income gap has a significant inhibitory effect on the total consumption of the whole society through marginal consumption propensity (Liu Yunzhuan, Song Yu, 2017) [40]. The second one is the substitution effect. As mentioned above, a small number of first-time companies to take the lead in improving production efficiency and high-quality products are the main sources of TFP improvement. At the same time, however, it should be noted that buyers from various income levels have significant differences in the effective demand for high quality products. Compared with the low-income group, whose consumption is usually concentrated on the basic products that maintain the basic needs of life, the high-income group has stronger willing and ability to consume high-quality products. Therefore, the widening income gap will stimulate the innovation behavior of the company by influencing the total consumption structure, and will ultimately induce the overall TFP level to rise. Zweimuller (2000) [21] discussed the theoretical mechanism by which the income gap drives technological progress through the consumption structure from the perspective of price effect. The above analysis shows that the income gap will have a “dual impact” on TFP through the income effect and substitution effect of consumption. That is to say, the expansion of the income gap will lead to the expansion of the income effect of consumption and the improvement of TFP on the one hand, and on the other, the substitution effect of consumption will not be conducive to the improvement of TFP.

### 3.3 Supply mechanism: Income effect and substitution effect

As mentioned earlier, the income gap will also affect TFP through the quality of labor supply. The widening income gap strengthens credit constraints and inhibits the ability of low-income groups to invest in human capital, which will undoubtedly have a negative impact on the improvement of labor quality. In the long run, it is not conducive to TFP improvement and economic growth (Galor, Moav, 2004; Chao Xiaojing, Shen Kunyu, 2014; Zhang Laiming, Li Jianwei, 2016) [32, 48–49]. The first is the income effect. The process of improving production efficiency is often accompanied by high-quality labor employment demand. Therefore, the improvement of TFP level in a country means the improvement of the overall quality of the domestic labor force. For example, Cheng Huifang and Lu Jiajun empirically using the panel data of large and medium-sized industrial enterprises in China from 1997 to 2010 to affirm the positive effect of human capital scale on TFP [50]. This view has been supported by many related studies (Sheng Yanchao, Zhou Yujiao, 2018) [51]. Therefore, the increases in income gap is not conducive to the human capital investment activities of low-income groups or to the expanding of human capital in a country, and will eventually limit the improvement of domestic TFP levels.

The second is substitution effect. More research examines the income effect of the income gap on human capital accumulation and considers that the income gap is linearly related to labor quality, which is obviously not objective. The production of high-quality products depends on high-efficiency workers, and the effect of financing constraints caused by the widening income gap on high-income groups is lower than that of low-income groups. This will stimulate high-income groups to invest in human capital for themselves and their family members, and further expand the human capital gap between high-income groups and low-income groups, which will promote high-income groups to play a more essential role in the production innovation processes. The increase in TFP level is due to the fact that some enterprises have increased their productivity first, so the labor supply substitution effect with widening income gap may have a positive impact on the innovation of enterprises.

After the logic deduction above, we can find that the income gap can have an “intermediary influence” on TFP through two channels: consumption and supply. While the impact mechanism can be further subdivided into income effects and substitution effects regardless of consumption or supply. The widening income gap on the one hand hinders the improvement of TFP through the income effect, and on the other hand promotes the increase of TFP through the substitution effect. It should be noted that the income effect and substitution effect of consumption and supply may have different aggregation effects on different income gap levels, and thus cause the nonlinear impact of income gap on TFP. That is, in the initial stage of low income gap, the expansion of income gap in one country will give priority to stimulating the high-quality total consumption and human capital investment.

**Fig 1:**
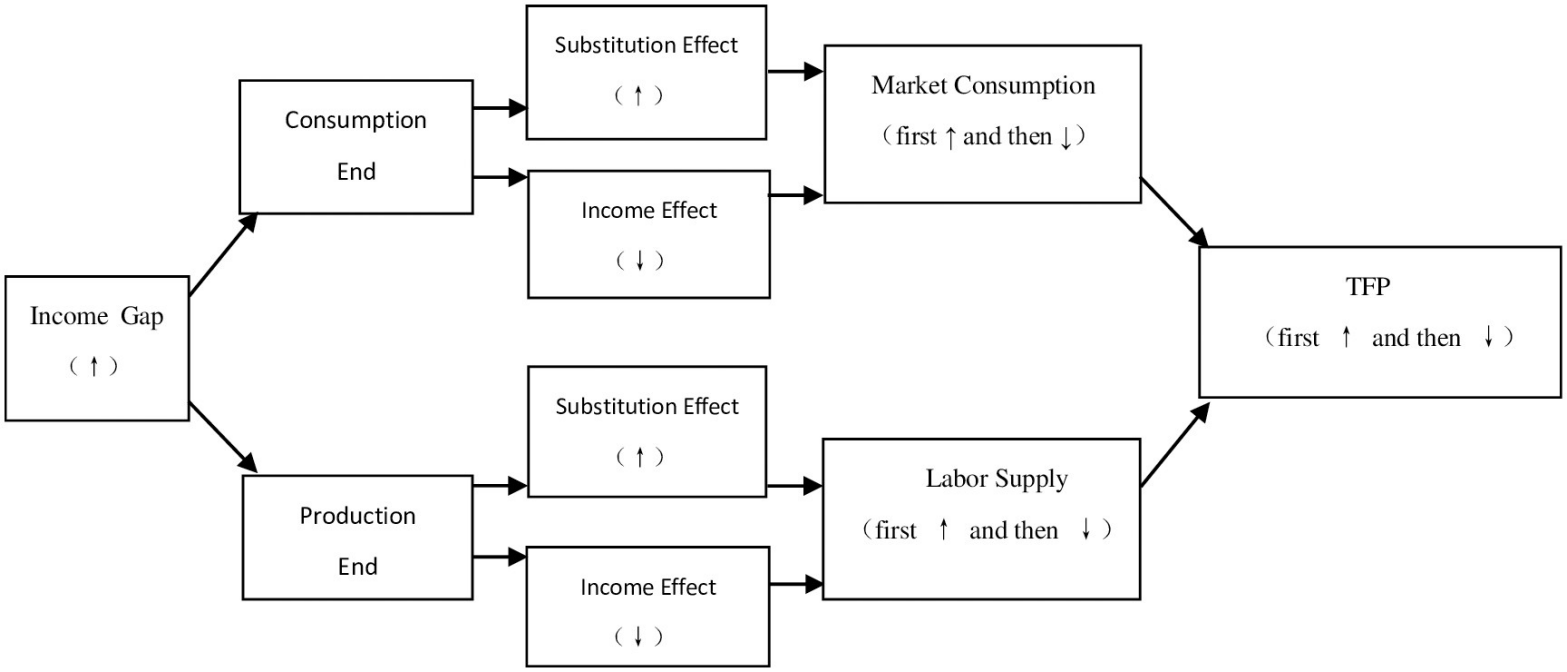
Technical roadmap.

At this time, the income effect of total consumption and labor supply has not been fully utilized. In this context, the substitution effect of the total consumption and labor supply (TFP promotion effect) with larger income gap is greater than the income effect (TFP hindrance effect), which is conducive to the improvement of domestic TFP. At the higher level of income gap, the continued widening income gap of a country may suppress the marginal tendency of high-income groups for high-quality total consumption and human capital investment, thus the substitution effect will continue to weaken. Meanwhile, the mechanism of expanding the size of the overall consumer market and strengthening the human capital investment constraints of low-income groups has hindered the growth of TFP. The income effect of total consumption and labor supply (TFP impediment effect) may in turn exceed the substitution effect (TFP promotion effect), which is obviously not conducive to TFP improvement. The above theoretical and logical deductions can be described by the following figure. The inverse relationship between the two mechanisms-the total consumption and labor quality- the income gap in a country continues to expand and the trend of total consumption and labor quality. As shown in Fig 1, it can be regarded that the effect of income gap on TFP is a non-linear inverted U-shaped curve shaped as “first rise and then fall”, and this inverted “U” hypothesis will become the logical basis of the empirical part of this paper.

## 4. Empirical methods and data

Due to changes in income effects and substitution effects, the income gap will have an impact on TFP through both market consumption and labor supply, and the impact is nonlinear. Therefore, this paper intends to use the following basic models for empirical research:

$${TFP}_{i,t}=\alpha +\beta_{1}{Gini}_{i,t}+\beta_{2}{Gini}_{i,t}^{2}+\gamma X_{i,t}+\varepsilon_{i,t}$$ (1)

According to Wang Jun and Liu Dong (2009), Gao Fan and Wang Yanan (2016), the lag item may “distort” and “absorb” the effect of income gap on TFP and eventually will “disturb” the actual impact of income gap on TFP[52, 9]. So, the explanatory variables in this paper will incorporate the lag term into the econometric model to explore the impact of a country’s income gap and other variables on the domestic TFP. In the above formula, TFP represents the total factor productivity of country i during period t; GINI represents the domestic income gap in country I; X represents other variables affecting TFP. Coefficient β_1_ and β_2_ represent the effects of the income gap and its squared term on TFP respectively. And when β_1_ is greater than 0 and β_2_ is less than 0, the income gap has an inverted U-shaped influence on TFP; γ indicates other factors affecting TFP. According to the theory of economic growth, in addition to the domestic income gap, TFP will also be affected by factors such as openness, infrastructure construction, inflation rate, energy consumption and other factors. Therefore, the formula (1) is extended as follows:

$${TFP}_{i,t}=\alpha +\beta_{1}{TFP}_{i,t-1}+\beta_{2}{Gini}_{i,t}+\beta_{3}{Gini}_{i,t}^{2}+\beta_{4}{EXP}_{i,t}+\beta_{5}{INS}_{i,t}+\beta_{6}{INF}_{i,t}+\beta_{7}{lnELE}_{i,t}+\varepsilon_{i,t}$$ (2)

In (2), EXPi, t, INSi, t, INFi, t, lnELEi, t respectively indicates the degree of openness, infrastructure construction, inflation rate and energy consumption, β_4_, β_5_, β_6_ and β_7_respectively indicate the influence of the above factors on TFP. Based on the previous description of the mechanism, the income gap affects TFP through two ways: market consumption and labor supply. These two approaches are subdivided into income effects and substitution effects affecting TFP. In this paper, CCi, t, CSi, t, LCi, t, LSi, t respectively represents the income effect of market consumption, the substitution effect of market consumption, the income effect of labor supply, and the substitution effect of labor supply. Therefore, we can write (2) as:

$${TFP}_{i,t}=\alpha +\beta_{1}{TFP}_{i,t-1}+\beta_{2}{CC}_{i,t}+\beta_{3}{CS}_{i,t}+{\beta_{4}{LC}_{i,t}+\beta_{5}{LS}_{i,t}+\beta }_{6}{EXP}_{i,t}+\beta_{7}{INS}_{i,t}+\beta_{8}{INF}_{i,t}+\beta_{9}{lnELE}_{i,t}+\varepsilon_{i,t}$$ (3)

In order to use the (2) and (3) formulas to examine the impact of domestic income disparity on domestic TFP, this paper intends to use the panel data of 53 countries from 1995 to 2014 for empirical measurement analysis. Positioning the time period from 1995 to 2014 has several purposes. The first is to better study the evolution of global production efficiency and regional income distribution after the globalization of production trade has deepened and the degree of world economic integration has entered a new stage. The second is to examine the impact of global economic transformation on regional income distribution after the global economic crisis in 1998 and 2008. And third, the availability of relevant data in 53 countries during this period is stronger. The variable research and data sources in this paper are as follows:

### 4.1 Total factor productivity (TFP)

Feenstra, Hanson used Diewert’s calculation method to re-measure the annual production efficiency of various regions of the world since World War II, and they took the 2011 national technical level of each country or region as 1[53]. The result represents the technical distance of the region to the United States in that period. This paper selects the production efficiency of the 53 major countries in the world from 1995 to 2014 as the explanatory variable data from the Penn World Table, version 9.0 database established by Feenstra et al. [54].

### 4.2 Income gap

The existing research mainly measures the income gap based on the per capita GDP (or per capita income) of different groups. Among them, Huang Xinfei et al. (2014) used the absolute value of per capita income difference to express the income gap, but this method lacks a reflection on the proportion of the population from different income level groups[55]; Li Renyu et al. (2015) reflect the characteristics of income distribution in the country based on the difference in income levels between the rich and the poor, but it is difficult to distinguish and explain the factors that cause income differentiation between the rich and the poor[56]. Hammar and Waldenström (2017) measured the Gini coefficient based on the Swiss Bank’s occupational survey income data and the ILO’s full population labor market statistics [57]. This method not only considers the impact of the proportion of the population from different income groups on the regional income gap, but also intuitively reflects the income disparity between groups of workers with different skills, which is in line with the main research of this paper. Therefore, this paper uses the Gini coefficient of each country published by Hammar and Waldenström to reflect the income gap.

In view of the fact that the original database collects statistics of the income gap every three years, this paper supplements the missing years according to the arithmetic average of two adjacent statistical years.

### 4.3 Control variable

In addition to the income gap, this paper attempts to control the following variables when studying the factors affecting TFP: the degree of openness (EXP) is expressed by the proportion of export trade in each region to the GDP of the country. The export volume and GDP of each region are derived from the World Bank database. Infrastructure (INS), expressed by the number of railway miles per unit of land in each country or region. The inflation rate (INF) is expressed as the annual inflation rate in each region. Existing studies have used regional highway mileage or hospital numbers to define the level of infrastructure in the region. However, given the scarcity of road mileage and hospital numbers in various countries, it will be difficult for us to find the above indicators. Therefore, this article has to use railway mileage as a proxy variable for the infrastructure. Energy consumption (ELE) is expressed in terms of per capita electricity consumption in each region. The above data are all from the World Bank database.

### 4.4 Mechanism variable

To elaborate the mechanism of the impact of income disparity on TFP, this paper intends to introduce the following variables:

1. The income effect of consumption (CC) is expressed by the real consumption per capita of each region which is calculated by deflating the per capita consumption expenditure according to the CPI index of each region (fixed base, 2011 = 1). Per capita consumption expenditure data is derived from the World Bank database and is in thousands of United States dollars. The reason why the per capita consumption expenditure is used to measure the total consumption income effect is that the expansion of the income gap will suppress the per capita consumption level by reducing the overall consumption propensity of the society. Some empirical studies have also confirmed this point (Feng Wei, Ban Jiatao, 2014) [44]. From the logical point of view, the expansion of the income gap will hinder the improvement of TFP through the income effect of total consumption. And the widening income gap will lower per capita consumption expenditure. Therefore, the sign of per capita consumption coefficient is expected to be positive.
2. The substitution effect (CS) of consumption is expressed by the Engel coefficient in each region. The Engel coefficient is equal to the per capita food expenditure divided by the per capita consumption expenditure. Per capita food expenditure data comes from the United Nations database. The reason why the Engel coefficient is used to measure the total consumption substitution effect is that the widening income gap will change the consumption structure of residents and lead to a decline in the Engel coefficient. Some empirical studies indicate that the income gap is negatively correlated with the Engel coefficient (Jiang Guogang, 2012; Zhang Lei et al. 2013) [58–59]. In the logical deduction of previous section, the expansion of the income gap will promote the improvement of TFP through the substitution effect of total consumption. And the expansion of the income gap will reduce the Engel coefficient. As a result, the symbol of the Engel coefficient is expected to be negative.
3. The income effect of the labor supply (LC) is expressed by the annual human capital level of each region. This paper uses the Human Development Index (HDI) to measure a country’s annual human capital level. The data comes from the UN database. The reason it is used to indicate the income effect of labor supply is that the expansion of the income gap will strengthen the human capital investment financing constraints, which is not conducive to the accumulation of human capital. Zhang Chewei shows that the widening income gap will lead to the “Matthew effect” of the return on education investment between groups with different income levels and will worsen human capital investment [60]. Therefore, the symbol of the human capital index coefficient is expected to be positive.
4. The substitution effect (LS) of labor supply is expressed by the illiteracy rate of each region. The illiteracy rate- equal to the illiterate population divided by the total population- can better reflect the tendency of low-income groups to invest in human capital to improve their working ability under different income gaps. The illiteracy rate data is from the Barrow and Lee Human Capital Database. The illiteracy rate is used to indicate the substitution effect of labor supply. This is because from the previous logical deduction, it can be found that the expansion of income gap may encourage low-skilled populations to improve their income levels by increasing human capital investment. The foregoing article theoretically clarifies that the expansion of the income gap will form a positive incentive for TFP through the substitution effect of labor supply, while the widening income gap will reduce the illiteracy rate. Therefore, the symbol of illiteracy rate coefficient is expected to be negative.

### 4.5 Selection of time range

In this paper, we have chosen a relatively long-time span which is 1995–2014. At the 1995 Madrid Summit, the European single currency was named as the “euro”, and the starting time for more relevant data was also 1995, so this article chose 1995 as the starting time; The CS and CC variables in the mechanism test mostly stopped in 2014 in the UNDATA and World Bank databases, so this article chose 2014 as the ending time. In summary, descriptive statistics of major variables are shown in Table 1.

**Table 1:** Granger-F test.

Null Hypothesis	Statistic F	P Value	Conclusion
Gini coefficient is not the Granger reason for the TFP index statistic	7.523	0.000	Refuse
Gini2 is not the Granger reason for the Gini coefficient statistic	8.074	0.000	Refuse

As can be seen from Table 1, the results of the Granger test indicate that the squared terms of the Gini coefficient and the Gini coefficient are both the Granger genesis of the TFP. Therefore, it is especially important to select the appropriate tool variables to solve the endogeneity problem of the original model. Therefore, this paper will try to solve the endogeneity problem of the model by using the GMM method.

## 5. Empirical analysis

**Fig 2:**
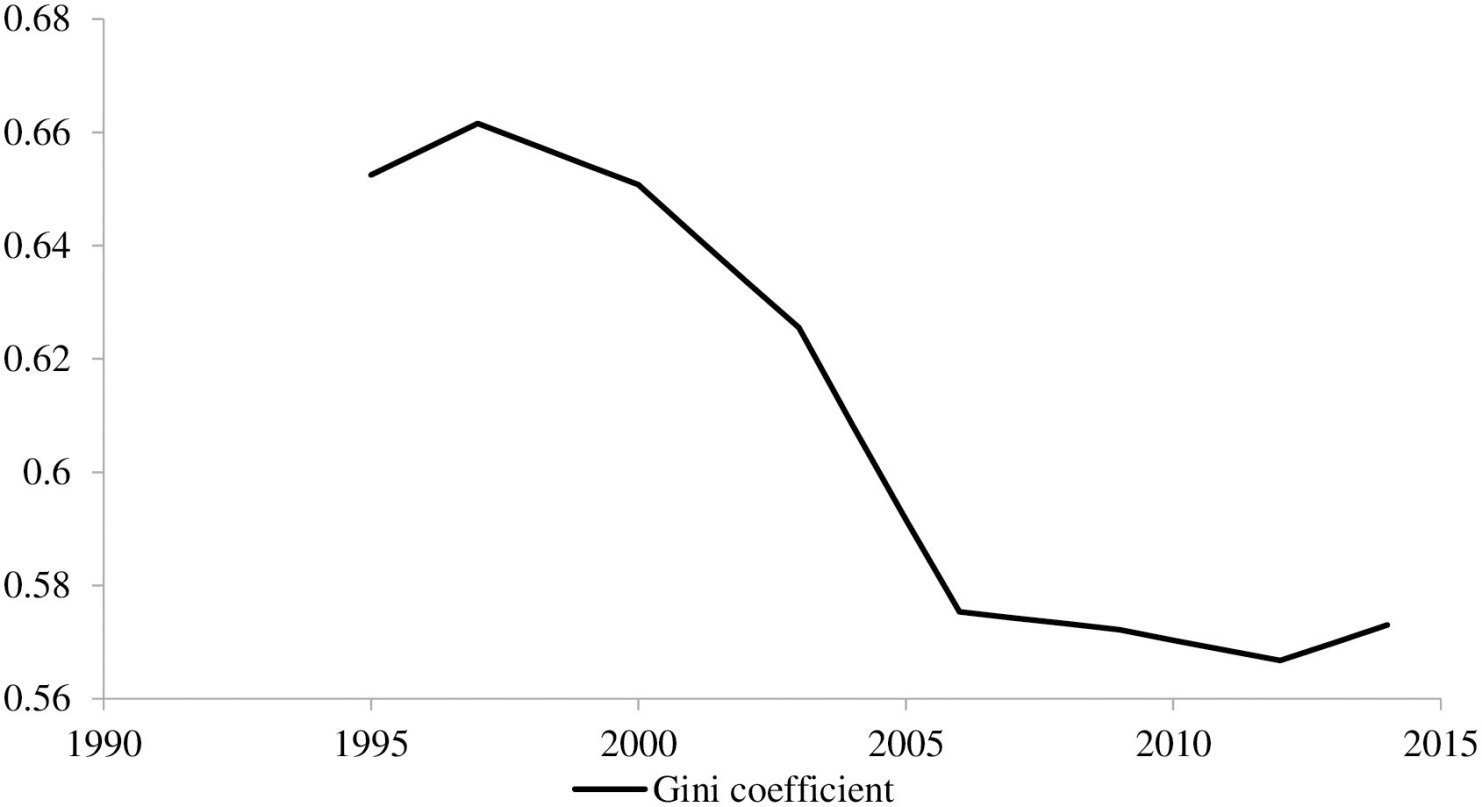
Changes of gini coefficient in the world from 1995 to 2014.

According to the world Gini coefficient measured by Olle Hammar and Daniel Waldenström (2017), between 1995 and 2014, the world income gap mainly experienced the “N-type”—three stages of “first rise, then fall and then rise” [57]. The specific detail is shown in Fig 2. The first stage was 1995–1997, in which the world Gini coefficient increased from 0.653 to 0.662, a growing range of more than 2.16%; the second phase was 1998–2012, and the world income gap experienced a long-term downward trend. The second stage was 1998–2012, and the world income gap experienced a long-term downward trend. During this period, the world Gini coefficient dropped from 0.658 to 0.567, with a total decline of more than 16%, and an average annual decline of more than 1%; the third stage is 2013–2014, when the world income gap has slowly expanded. It should be noted that the world Gini coefficient has shown a downward trend in 1998 and around 2008. This may be because during the economic development period, developed countries have widened the income gap between their own countries and developing countries through comparative advantages in economic development. While in the recession period, developed countries with higher degree of marketization and higher openness have received more shocks. This has instead improved the global income imbalance situation. Jiang Tao and Zhao Wenlong also supported this view [61].

The descriptive statistics of model variables are shown in Table 2:

**Table 2:** Descriptive statistics of major variables.

Variables	Observation	Mean	Standard Deviation	Minimum	Maximum	Skewness	Kurtosis
TFP	1060	0.97	0.10	1.33	0.53	-1.34	3.49
G	1060	0.31	0.10	0.61	0.12	0.65	-0.35
CC	1060	0.17	0.21	4.03	0.05	10.78	163.51
CS	1060	2.70	1.49	9.41	-0.80	1.33	5.12
LC	1060	-0.24	0.14	-0.06	-0.80	-1.36	1.92
LS	1060	0.045	0.065	0.458	0.000	3.042	11.98
EXP	1060	0.46	0.34	2.31	0.07	2.68	9.11
INS	1060	0.03	0.03	0.12	0.00	1.04	0.18
INF	1060	0.08	0.34	9.59	-0.16	22.41	591.98
lnELE	1060	8.35	0.98	10.15	4.66	-1.23	2.09

Data are collated by authors.

Since the model is highly endogenous, this paper adds the lag term of TFP to the econometric model as an explanatory variable. In addition, in view of the strong correlation between the illiteracy rate of explanatory variables and HDI, the income effect and substitution effect of labor supply in the original mechanism analysis model are respectively regressed to avoid collinearity. The modified model is as follows:

$${TFP}_{i,t}=\alpha +\beta_{1}{TFP}_{i,t-1}+\beta_{2}{CC}_{i,t}+\beta_{3}{CS}_{i,t}+{\beta_{4}{LC}_{i,t}++\beta }_{5}{EXP}_{i,t}+\beta_{6}{INS}_{i,t}+\beta_{7}{INF}_{i,t}+\beta_{8}{lnELE}_{i,t}+\varepsilon_{i,t}$$ (4)

and,

$${TFP}_{i,t}=\alpha +\beta_{1}{TFP}_{i,t-1}+\beta_{2}{CC}_{i,t}+\beta_{3}{CS}_{i,t}+{\beta_{4}{LS}_{i,t}++\beta }_{5}{EXP}_{i,t}+\beta_{6}{INS}_{i,t}+\beta_{7}{INF}_{i,t}+\beta_{8}{lnELE}_{i,t}+\varepsilon_{i,t}$$ (5)

Since there may be a “reciprocal causation” between the interpreted variable and the explanatory variable. Therefore, based on (4) and (5), the bidirectional fixed effect model is used to investigate the effect of Gini coefficient on the inverted U shape of TFP. The empirical results are shown in Table 3:

**Table 3:** Analysis results of full-sample model.

Variables	Inflection point determination			Mechanism analysis			
	(1)	(2)	(3)	(4)	(5)	(6)	(7)
	(1)	(2)	(3)	(4)	(5)	(6)	(7)
TFP_t-1_	0.915***	0.902***	0.811***	0.889***	0.895***	0.887***	0.892***
	(80.31)	(77.12)	(15.22)	(78.89)	(69.70)	(69.57)	(68.27)
Gini	0.283***	0.223***	1.365*				
	(3.83)	(2.68)	(1.71)				
Gini^2^	-0.527***	-0.428***	-2.509**				
	(-4.46)	(-3.62)	(-2.06)				
CC				0.039***	0.046***	0.035***	0.041***
				(4.38)	(5.76)	(3.58)	(4.29)
CS				-0.015**	-0.013**	-0.016**	-0.015**
				(-2.35)	(-2.14)	(-2.57)	(-2.37)
LC				0.106***		0.081*	
				(2.82)		(1.95)	
LS					-0.117***		-0.089**
					(-3.12)		(-1.99)
EXP		0.017*	-0.002			0.019**	0.021**
		(1.82)	(-0.05)			(2.03)	(2.24)
INS		0.202	0.581			0.415	0.255
		(0.78)	(1.42)			(1.58)	(0.96)
INF		-0.002	0.001			-0.001	-0.001
		(-0.92)	(0.21)			(-0.34)	(-0.40)
lnELE		0.032***	-0.041*			0.014*	0.012
		(5.06)	(-1.79)			(1.66)	(1.42)
Constant	0.050***	-0.208***	-	0.044	-0.003	-0.087	-0.109*
	(2.62)	(-3.93)	(-)	(1.64)	(-0.19)	(-1.26)	(-1.75)
Hansen test			0.153				
N	1007	1007	1007	1007	1007	1007	1007
Adj.R^2^	0.151	0.9716	-	0.7464	0.7248	0.9185	0.9185

The above table gives the regression results of STATA13.1. As far as the determination of the inflection point of the world is concerned, the adjusted coefficient of judgment is 0.9716, indicating that the regression result is relatively good, and the explanatory variable has a strong interpretation of the TFP. For the control variables, the coefficients of EXP, INS, INF, and lnELE are 0.017, 0.202, -0.002, and 0.032, respectively, but the statistical results of INS, and INF are not significant. It shows that TFP is not sensitive to infrastructure construction and inflation rate, but the degree of openness and energy consumption has a positive effect on TFP. The coefficients of the explanatory variables Gini and Gini^2^ are 0.283 and -0.527, respectively, and are statistically significant. This shows that for every unit of income gap increase, it will lead to an increase of 0.283 units of TFP on the one hand, and on the other, it will cause the TFP growth rate to drop by 0.527 units. Besides, the endogenous test results in this paper are shown in column 3 of the Table 3. The results of column (3) show that: First, the Hansen test of overid is about 0.153, indicating that the selection of the instrumental variables is valid; Second, the coefficient significance and symbol of the Gini coefficient and its quadratic term are still consistent with the original model regression results after controlling the endogeneity of the model. Therefore, from the above conclusions, it can be considered that the original model regression results are robust.

**Fig 3:**
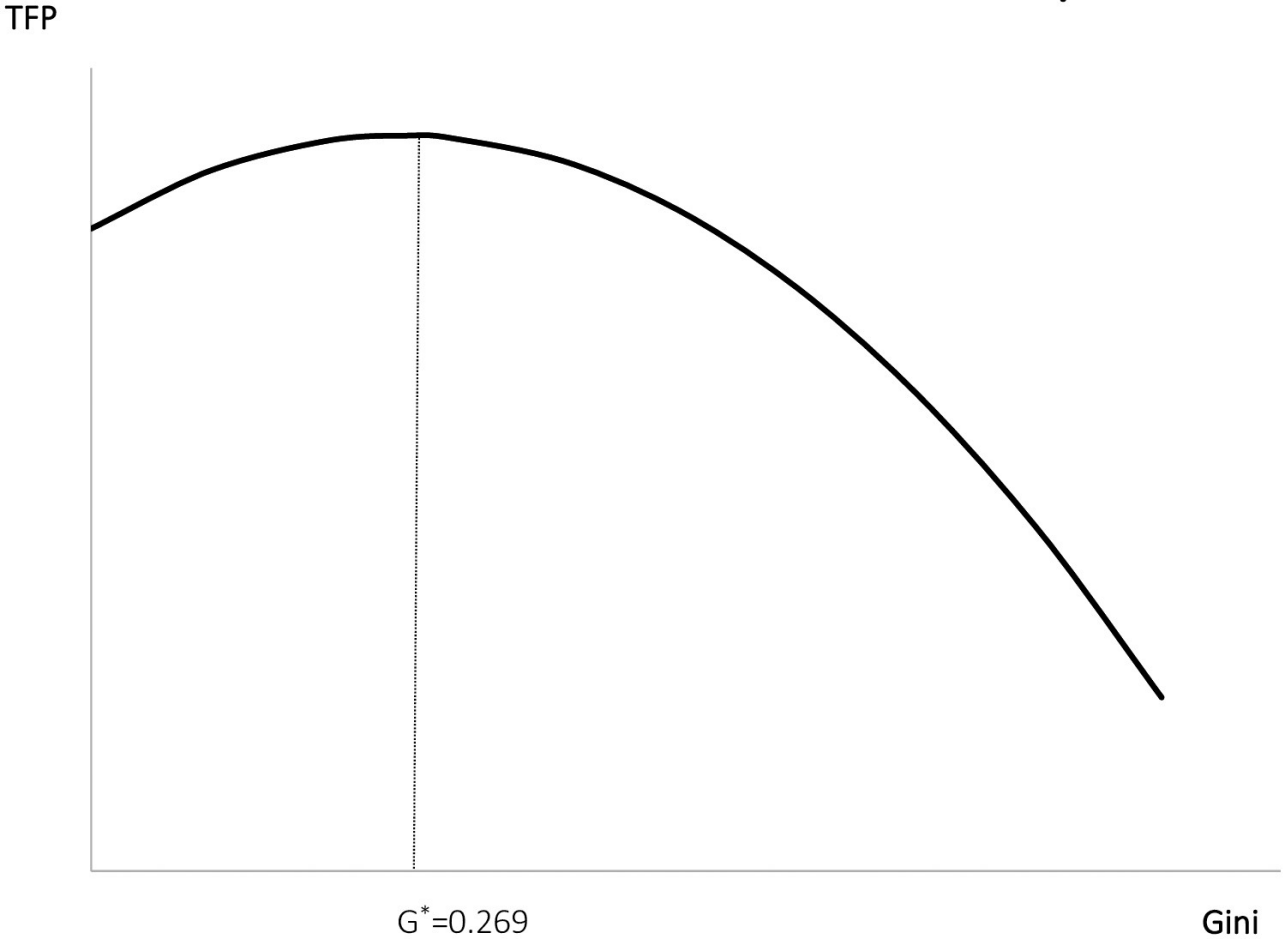
The “Inverted U” effect of income gap on TFP.

In conclusion, the effect of income gap on TFP has indeed experienced the inverted U-type change process of “first rise and then fall”. If we find the first derivative of the explanatory variable Gini of the regression equation and calculate its extreme point, as shown in Fig 3, we can conclude that the inverted "U" type inflection point appears roughly in the income gap of 0.269. The result is obviously of great economic significance. The average Gini index of income disparity in each region during the inspection period was 0.307, indicating that the world’s overall income gap is still located to the left of the inverted U-shaped inflection point. In other words, a modest expansion of the income gap may have a positive effect on TFP.

The domestic Gini coefficient of China from 1995 to 2014 is shown in Table 4:

**Table 4:** Gini coefficient in China from 1995 to 2014.

Year	Gini	Year	Gini
1995	0.312	2005	0.387
1996	0.326	2006	0.307
1997	0.341	2007	0.317
1998	0.367	2008	0.327
1999	0.392	2009	0.337
2000	0.418	2010	0.306
2001	0.461	2011	0.276
2002	0.505	2012	0.245
2003	0.548	2013	0.247
2004	0.468	2014	0.248

It could be found that the domestic Gini coefficient of China is located to the right of the inflection point of the inverted U-shaped curve before 2012 and to the left after 2012. This shows that the expansion of the income gap is not conducive to China’s TFP before 2012, but is conducive after 2012.

Besides, the income gap has an inverted U-type effect on TFP. To test the internal mechanism of the impact, a mechanism analysis model can be used for empirical research. As shown in the above table, the adjusted coefficients of the full-sample mechanism analysis model both are 0.9185, respectively, indicating that the regression result is good, and the explanatory variables have strong interpretation of the TFP explained. Among the control variables, the coefficients of EXP, INS, INF, and lnELE in Model 5 are 0.019, 0.415, -0.001, and 0.032, respectively. INS and INF are not statistically significant, but the degree of openness and energy consumption are both significant. The EXP, INS, INF and lnELE coefficients in Model 6 were 0.021, 0.255, -0.001, and 0.014, respectively, except for the degree of openness, which was statistically significant, the other variables were not significant. The coefficients of CC and CS in Model 5 and Model 6 were 0.046 and -0.013, 0.035 and 0.016, respectively, and were statistically significant. The LC coefficient in Model 5 was 0.081 and significant, and the LS coefficient in Model 6 was -0.089 and significant. The CS and LS symbols are negative, indicating that the higher the Engel coefficient, the more obvious the inhibition of TFP, and the illiteracy rate also has a negative impact on TFP growth. The widening income gap will stimulate TFP growth by changing the structure of the labor supply structure. The CC and LC symbols are positive, indicating that per capita consumption has a promoting effect on TFP, and the widening income gap is not conducive to improving the overall scale of the market and thus deteriorating the TFP. Meanwhile, the human capital index has a promoting effect on TFP, and the expansion of the income gap is not conducive to the overall improvement of the labor level and thus inhibits the growth of TFP. The above empirical results show that the income gap between countries and regions in the world affects TFP through two ways: market consumption and labor supply. Both of these approaches can be subdivided into two totally different effects-income effects and substitution effects. Therefore, the empirical research results strongly support the logical deduction of the previous article.

To further test the theoretical model, this paper subdivides 53 sample countries into developing countries (17), medium-developed countries (12), and developed countries (24), as shown in Table 5. Since the panel data used in the model has a limited time span, the group empirical model is changed to:

**Table 5:** Types and classification of countries.

Type	Countries
Low-income countries	Brazil, Bulgaria, China, Colombia, India, Indonesia, Iran, Kenya, Malaysia, Mexico, Philippines, Russia, South Africa, Thailand, Turkey, Ukraine, Venezuela
High-income countries	Chile, Cyprus, Czech, Estonia, Hungary, Latvia, Lithuania, Poland, South Korea, Saudi Arabia, Slovakia, Slovenia
Top-income countries	Australia, Austria, Belgium, Canada, Denmark, Finland, French, Germany, Greece, Ireland, Israel, Italy, Japan, Luxemburg, Netherlands, New Zealand, Norway, Portugal, Singapore, Spain, Sweden, Switzerland, UK, USA

Data are collated by the author.

$${TFP}_{i,t}=\alpha +\beta_{1}{TFP}_{i,t-1}+\beta_{2}{Gini}_{i,t}++\beta_{3}{EXP}_{i,t}+\beta_{4}{INS}_{i,t}+\beta_{5}{INF}_{i,t}+\beta_{6}{lnELE}_{i,t}+\varepsilon_{i,t}$$ (6)

The group empirical results are shown in Table 6:

**Table 6:** Analysis results of sub-sample model.

Variables	Interpreted variables TFP_t_								
	Direction determination			Mechanism analysis					
	TFP_low_	TFP_high_	TFP_top_	TFP_low_	TFP_high_	TFP_top_	TFP_low_	TFP_high_	TFP_top_
	(7)	(8)	(9)	(10)	(11)	(12)	(13)	(14)	(15)
TFP_t-1_	0.867***	0.942***	0.893***	0.849***	0.849***	0.934***	0.784***	0.847***	0.848***
	(34.44)	(44.78)	(44.76)	(30.57)	(29.86)	(40.13)	(17.96)	(37.13)	(37.58)
Gini	-0.102***	-0.123**	0.059**						
	(-2.92)	(-2.61)	(1.99)						
CC				0.041**	0.040**	0.046	0.080***	0.047***	0.048***
				(2.37)	(2.21)	(1.63)	(3.08)	(2.76)	(2.82)
CS				-0.012	-0.013	0.002	-0.195	0.714***	0.721***
				(-1.49)	(-1.50)	(0.01)	(-1.45)	(4.28)	(4.26)
LC				0.094		0.009		0.025	
				(1.08)		(0.08)		(0.33)	
LS					0.009		2.733***		-0.023
					(0.11)		(4.00)		(-0.42)
EXP	-0.035	0.065***	0.017*	-0.052**	-0.048*	0.073***	0.110***	0.024***	0.024***
	(-1.37)	(2.98)	(1.71)	(-1.99)	(-1.83)	(3.24)	(4.71)	(2.41)	(2.57)
INS	-2.864	0.440	0.435***	-1.132	-1.711	0.654	0.534	0.305	0.259
	(-0.42)	(0.65)	(2.28)	(-0.42)	(-0.65)	(0.92)	(0.80)	(1.56)	(1.23)
INF	0.002	-0.017	0.077*	0.020	0.002	-0.016	-0.110	0.035	0.034
	(0.50)	(-0.47)	(1.84)	(0.67)	(0.65)	(-0.43)	(-0.30)	(0.87)	(0.84)
lnELE	0.046***	0.033*	0.009	0.026	0.037**	0.013	0.067***	0.003	0.003
	(3.49)	(1.84)	(0.67)	(1.52)	(2.24)	(0.65)	(2.86)	(0.18)	(0.23)
Constant	-0.128	-0.211	-0.017	-0.028	-0.147	-0.212	-0.661***	-0.116	-0.128
	(0.143)	(-1.40)	(-0.14)	(-0.20)	(-1.38)	(-1.39)	(-3.58)	(-0.75)	(-0.96)
N	323	228	456	323	323	228	228	456	456
Adj.R^2^	0.7907	0.9248	0.8491	0.7556	0.7492	0.7572	0.4893	0.9100	0.8723

Bootstrapped standard errors are reported in parenthesis.

***, ** and * refers to significant at 1%, 5% and 10% respectively

According to group empirical research, it can be found that the Gini parameter coefficients of developing countries, medium-developed countries and developed countries are -0.102, -0.123 and 0.059, respectively, and they are statistically significant. It shows that if the domestic income gap is widened, it can promote the growth of TFP in developed countries, but will inhibit the TFP of developing countries and medium-developed countries, and its effect on TFP growth in developing countries is stronger than that in medium-developed countries. From the perspective of the impact mechanism, in developing countries, CC is significant but CS, LC, and LS are not significant, indicating that in developing countries, per capita consumption can effectively promote domestic TFP growth while labor supply has limited impact on TFP. In other words, the market consumption income effect has a strong explanatory power for TFP changes. In the medium-developed countries, LS is statistically significant, which indicates that the differentiation of the labor force in the country may contribute to the improvement of TFP to a certain extent. This is because the technology gap between these countries and developed countries is shrinking, making it increasingly difficult to introduce and absorb foreign advanced technologies, which may force them to concentrate their own limited resources on their own research and development to achieve technological breakthroughs in certain areas, and this will lead to the horizontal division of their domestic labor force. In developed countries, CC and CS are significant while LC and LS are not, indicating that market consumption has a significant impact on TFP. The reason may be related to the idea that when the economy develops to a higher stage, the growth may depend on consumption. And the labor supply has limited impact on TFP. Possible reason is that the human capital level in developed countries has been relatively high for a long time. So, further improvement of human capital level cannot significantly improve the existing production efficiency of the country. Among the control variables, the degree of openness has a significant impact on the TFP of each sample, and it has a negative effect on the promotion of TFP in developing countries. This can be explained by the idea that in the global trade environment, the huge gap of technology level between the developing countries and the developed countries makes it difficult for the technological output of developed countries to be learned and absorbed by developing countries, which leads to the “low-end locking” of the technological level of developing countries, thus inhibiting the growth of TFP in these countries. For the medium-developed countries and developed countries, opening up to the outside world will strengthen their production as well as their middle and high position in the industrial chains, which is obviously beneficial to the domestic TFP promotion. The above empirical results show that the income gap has different effects on TFP in countries with different economic development levels. And the effect and direction of the income effect and substitution effect through market consumption and labor supply are quite different. The previous theoretical deduction has been further supported by empirical research. Further, the above table does not consider the interaction between the income gap and the impact mechanism. For example, the income gap is an explanatory variable of the structure (or scale) effect of consumption (or labor). They will have different degrees of influence on countries and regions at different stages of development. To this end, the following table further considers the impact of the income gap and the interaction mechanism to analyze the difference in the influence of the structure (or scale) effect of consumption (or labor).

## 6. Conclusion

Technological progress and the narrowing of the domestic income gap are important issues for the balance of global development nowadays. This paper explores the ways and mechanisms of the impact of income disparity on TFP, and elaborates from the theoretical level: the income gap affects TFP through both market consumption and labor supply, and each of these influences has its own income effect and substitution effect, which causes the TFP to exhibit the inverted U-shaped nonlinear trend of “first increase and then decrease.” Based on the above theoretical logic, this paper uses the panel data of 53 countries and regions in the world from 1995 to 2014 for empirical research. The results show that the domestic income gap does cause the TFP to have an inverted “U”-type nonlinear trend of “first increase and then decrease”. The inflection point of TFP from growth to decrease appears in the domestic income gap of 0.269. In addition, the income gap does have an impact on TFP through both market consumption and labor supply.

The paper proposes policy recommendations to promote the growth of world TFP: First, in addition to the conventional factors, countries and regions must pay attention to the impact of the domestic income gap in the process of formulating TFP growth policies. Since the impact of the income gap on TFP is non-linear, the traditional idea that income gap widens or shrinks the linear improvement of TFP has flaw in terms of theoretical logic, and it may not be scientific to formulate policies through this traditional idea. Therefore, it is more plausible to distinguish and measure the impact of the income gap on TFP in a specific time period.

Second, the inverted U-shaped curve inflection point probably appears at the income gap of 0.269. When the developing countries and the moderately developed countries as a whole are in the stage of expanding the income gap, the larger income gap will inhibit the development of their domestic TFP growth. And the developed countries are generally in the development stage where the expansion of the income gap will promote the growth of domestic TFP. Therefore, narrowing the domestic income gap between developing and the moderately developed countries is conducive to improving domestic TFP. While appropriately expanding the domestic income gap in the developed countries will help to improve their TFP.

Third, China, as the largest developing country in the world, has its Gini coefficient on the right side of the inverted U-shaped curve inflection point before 2012. And after 2012, it appears on the left side of the inverted "U" curve inflection point, which means that the traditional income gap reduction measures may have a very limited improvement effect on the current Chinese TFP. In view of the fact that China’s Gini coefficient is generally near the turning point in recent years (that is, the income gap has the strongest effect on domestic TFP), simply expanding or narrowing the income gap will inhibit domestic TFP. On the one hand, China should deepen the marketization of labor income distribution, promote a “more pay for more work” distribution system and improve the tax system to expand middle-income groups; On the other hand, it is necessary to adhere to the established policy of “precise poverty alleviation”. Reduce the cost constraint of the transition from unskilled labor to skilled labor by improving the public expenditure system and promoting “point-to-point” poverty alleviation measures for low-income groups. These measures will be particularly important to promote technological innovation and foster new economic drivers. These measures will be particularly important to promote technological innovation and foster new economic drivers.

## Supporting information

S1 Database (ZIP)
